# Authors’ Reply: Japan's Telepsychiatry Dissemination: Current Status and Challenges

**DOI:** 10.2196/69798

**Published:** 2025-01-20

**Authors:** Yuka Sugawara, Yosuke Hirakawa, Masao Iwagami, Haruo Kuroki, Shuhei Mitani, Ataru Inagaki, Hiroki Ohashi, Mitsuru Kubota, Soichi Koike, Rie Wakimizu, Masaomi Nangaku

**Affiliations:** 1 Division of Nephrology and Endocrinology The University of Tokyo Tokyo Japan; 2 Department of Health Services Research Institute of Medicine University of Tsukuba Ibaraki Japan; 3 Clinic Pauroom Tokyo Japan; 4 Department of Education College of Education, Psychology and Human Studies Aoyama Gakuin University Tokyo Japan; 5 TAMA Family Clinic Tokyo Japan; 6 Department of General Pediatrics and Interdisciplinary Medicine National Center for Child Health and Development Tokyo Japan; 7 Division of Health Policy and Management Center for Community Medicine Jichi Medical University Tochigi Japan; 8 Department of Child Health and Development Nursing Institute of Medicine University of Tsukuba Ibaraki Japan

## Introduction

We thank the authors for their knowledgeable comments [1] on our study [2]. Our results suggest that although telemedicine is not widely used in the field of psychiatry and psychosomatic medicine, it is desired by the patient population receiving medical care. Deregulation of the restriction and setting higher reimbursement rates are considered important, but evidence is needed for this to happen. We believe that the results recently presented by Dr Kishimoto and colleagues [3] are very important, and the fact that they show that telemedicine in this field is not inferior to face-to-face care is good information not only for health care providers and the patient population, but also for government agencies as a resource for developing policy. This kind of evidence needs to be accumulated, and more research on telemedicine is needed in the future.
